# Research on lower extremity health in patients with multiple sclerosis: a systematic scoping review

**DOI:** 10.1186/s13047-020-00423-x

**Published:** 2020-08-27

**Authors:** Minna Stolt, Anne-Marie Laitinen, Juhani Ruutiainen, Helena Leino-Kilpi

**Affiliations:** 1 Department of Nursing Science, University of Turku, 20014 Turku, Finland; 2 Turku University Hospital, Turku, Finland; 3 Finnish Neuro Society, Masku, Finland; 4 Department of Neurology, University of Turku, Turku, Finland

**Keywords:** Multiple sclerosis, Lower extremity, Research, Review

## Abstract

**Background:**

Multiple sclerosis (MS) often affects ambulation and the function of the lower limbs. However, little is known about how much research has been conducted on lower extremity health in patients with MS.

**Objective:**

To analyse empirical studies and their evidence on lower extremity health in patients with MS, in order to identify the need for future studies in key areas.

**Methods:**

A systematic scoping review was conducted. A literature search of Medline (PubMed), CINAHL (EBSCO) and the Cochrane Library databases was performed. The search covered the period up to 15 January 2020 from the earliest records available. This led to the inclusion of 42 empirical articles. The data were analysed using content analysis and quantification techniques.

**Results:**

The research on lower extremity health focused primarily on two main areas: gait and lower extremity muscle strength. Lower extremity health was assessed using a variety of methods, most of which consisted of objective physical tests and gait analysis. Patients with MS had many problems with the health of their lower extremities, which manifested in walking difficulties, balance problems, muscle weaknesses and spasticity. In the feet, pes cavus, claw toes, oedema and altered foot sensation were common.

**Conclusions:**

MS affects lower limb and foot health, and these problems can affect patients’ daily lives. However, the extent of these problems is poorly understood, partly due to the dearth of research on lower limb and foot health. Therefore, further research is warranted in order to better understand the impact of MS on foot and lower limb health in everyday life.

## Introduction

Multiple sclerosis (MS) is an immune-mediated disease with a wide variation in its clinical course. Most patients with MS are initially diagnosed with a relapsing-remitting form of the illness, and the progression usually begins at around 40 years of age [1]. It is estimated that in 2016, there were more than 2.2 million prevalent cases of MS worldwide [2]. The highest estimates on prevalence per 100,000 people were for North America (167) and Western Europe (127) [2]. In a recent Finnish registry study, the mean age of patients with a new diagnosis of MS was 37.0 years (range: 15–69) and the female/male ratio was 2.6 [3].

Spasticity and weakness in the lower extremities are among the most common signs and symptoms of MS. As such, the validated Expanded Disability Status Scale (EDSS), [4] the most commonly used rating scale for disability in MS, is heavily based on the motor function of the lower limbs. Ambulatory impairment related to MS is a major factor in reducing patients’ quality of life and their ability to perform daily activities. Approximately 75% of patients with MS have limitations with walking [5] and balance [6–8]. Using prospective measures, fall rates of 56% have been reported in a recent meta-analysis of 537 individuals, with 37% of the study population falling recurrently [9]. Moreover fear of falling is associated with recurrent falls in patients with MS [10] thus impacting mobility and independent living. Also external factors like poorly fitting footwear increases risk for falls in patients with MS [11].

Investigations into the functional ability of patients with MS have focused on walking, postural control [7–9, 12] and gait abnormalities [13, 14]. Interventions including physical exercise, orthotic therapy and electrical stimulation have been shown to decrease the risk of falls, [15–17] although the evidence regarding the effects of these interventions is sparse and uncertain [18]. However, the majority of the research seems to focus on the musculoskeletal system as a whole, rather than the lower extremities in particular. In this review, ‘lower extremity health’ refers to the structure and functions of the lower extremities from the hips to the toes.

With regard to the lower extremities, muscle strength, optimal alignment of the joints, and foot and ankle stability form the basis for safe walking and motion [19]. However, despite the importance of lower extremity health, many persons have foot problems. In the general population, foot pain, [20, 21] hallux valgus, [22, 23] flat foot [24] and skin and nail problems [25] are the most common issues. Lower extremity health and the related problems have been considered in many patient groups with long-term health conditions such as diabetes, [26, 27] rheumatoid arthritis [28] and lupus [29]. For example, patients living with diabetes have an increased risk of developing foot problems such as neuropathic ulcers [30]; therefore, the number of foot problems in diabetic patients is higher than in the general population [31].

However, there seems to be a paucity of existing reviews examining foot problems in patients with MS.

Foot problems can add to a patient’s level of disability by causing changes in the structure and function of the body. Functional loss in the hips, knees, ankles or feet significantly increase the risk of falls, especially in older people [32]. Toe deformities, such as hallux valgus, hammer toe and claw toes, reduce the area used for balance in the sole of the foot, increasing postural sway and risk of falls [32, 33] and decreasing gait velocity [34]. In addition, reduced medial arch height and forefoot disorders (such as splay foot) change the kinematics and muscle activation of the foot, leading to altered gait patterns [35, 36]. Prolonged foot pain is also a strong risk factor for falls [37] and is associated with functional limitation, resulting in challenges conducting activities of daily living [38]. Among patients with MS, foot drop symptom where dorsiflexion of the ankle joint is reduced during gait is common [39]. Foot drop leads to poor foot clearance during gait and significantly elevates the risk of trips and falls [39]. Overall, foot problems negatively impact daily life and reduce quality of life [20, 40].

Although it is understood that MS causes ambulatory impairment, little is known about lower extremity health of those living with the disease. Investigations into lower extremity health will create opportunities to develop interventions that support functional ability. The research on lower extremity health among patients with MS seems to be fragmented, however, and a thorough synthesis of the research evidence is lacking. Therefore, a systematic scoping review was conducted in order to identify the potential size and scope of the available research evidence on lower extremity health in patients with MS.

### Aim

The aim of this review was to analyse empirical studies and their evidence on lower extremity health in patients with MS, in order to identify the need for future studies in key areas.

The following research questions were formulated:

1. What is the focus of lower extremity health research in patients with MS?
2. What methods have been used to study lower extremity health in patients with MS?
3. What are the main lower extremity problems in patients with MS?

## Methods

A systematic scoping review [41] was conducted. The Preferred Reporting Items for Systematic Reviews and Meta-Analyses (PRISMA) extension for scoping reviews [42] was used to outline this review. The review followed predetermined, unpublished, protocol.

### Literature search

A literature search was performed in three international scientific databases – Medline (PubMed) and CINAHL (EBSCO) – and the Cochrane Library. The search covered the period between 15 January 2020 and the earliest records available: from 1966 for Medline/PubMed, from 1988 for CINAHL and from 1992 for the Cochrane Library. Medical Subject Headings (MeSH terms, in Medline and Cochrane Library) and Major Headings (in CINAHL) were used to identify studies focusing on MS. Multiple search terms were used in order to cover the topic of lower extremity health as widely as possible. The final search sentence was as follows: Multiple sclerosis[MeSH] AND (foot OR feet OR “lower extremity” OR “lower extremities” OR “lower leg” OR “lower legs” OR “lower limb” OR “lower limbs”) AND (health OR problem* OR disorder* OR complaint* OR deformit* OR disabilit* OR condition*). The search was limited to title and abstract levels and to studies published in English. The literature search produced a total of 446 hits (*n* = 387/Medline, *n* = 56/CINAHL, *n* = 3/Cochrane).

### Study retrieval process

**Fig. 1:**
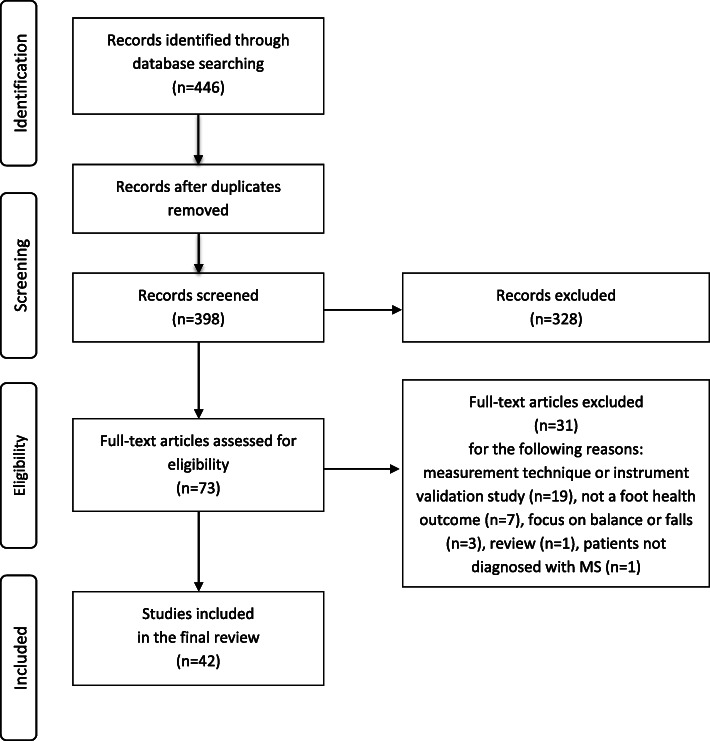
Data extraction flowchart

The studies were assessed against predetermined eligibility criteria (Table 1). The study retrieval process was carried out by two researchers (A-ML, MS) in two phases (Fig. 1). First, the titles and abstracts of the studies were screened against the inclusion and exclusion criteria. The researchers worked independently and discussed their choices before the next phase. In the case of a disagreement, this was discussed and a consensus was achieved within the research team. In total, 328 studies were excluded because they did not focus on lower extremity health or they were instrument-development or instrument-validation studies. The remaining 73 studies were included in the second phase, which entailed a full inspection of the text. After careful reading of the full texts and achieving a consensus, 31 studies were excluded. This resulted in 42 studies being included in the review and the final analysis.

**Table 1:** Eligibility criteria

Inclusion criteria:	
• empirical study	
• adult patients with MS as research informants	
• focus on foot or lower extremity health or functions or on walking (measured using non-invasive methods)	
Exclusion criteria:	
• review or theory articles	
• focus on the outcomes of foot or lower extremity surgery	
• pharmacological studies or used only invasive methods, such as electrical stimulation • instrument-validation, feasibility or case studies

### Data extraction and analysis

For the data extraction, detailed information from the studies were collected and entered on a data extraction spreadsheet. The information consisted of the following: author names, year of publication, country of origin, study aim, study setting, study design, methods of data collection and analysis, participants (sample size, mean age), and main results. The data were analysed using content analysis and quantification. The original expressions in the studies were used, which removed the need for any interpretation.

### Critical appraisal of individual sources of evidence

Critical appraisal of included studies was performed using the Mixed Methods Appraisal Tool (MMAT) [43] by two authors (MS, A-ML). The MMAT is applicable for empirical studies using different research designs namely qualitative research, randomized controlled trials, non-randomized studies, quantitative descriptive studies, and mixed methods studies. Each study was evaluated against five items focusing on methodological quality of the study with response scale yes/no/can’t tell.

## Results

### Description of the studies

The studies were published between 1978 and 2019, predominantly in the 2010s (*n* = 32). They were published in the United States (*n* = 15), Italy (*n* = 5), Australia (*n* = 2), Denmark (*n* = 2), Israel (*n* = 2), Romania (*n* = 2), Switzerland (*n* = 2), Turkey (*n* = 2) and the United Kingdom (UK) (*n* = 2). In each of the following countries, one study was published: Belgium, Brazil, Finland, France, Greece, Qatar and Spain. In addition, one study [44] included participants from five countries: France, Germany, Italy, Spain and the UK. The number of participants in each study ranged from 8 to 2171 (mean 97, median 29, SD 335). Only four of the studies included more than 100 subjects. All of the participants were patients who had been diagnosed with MS, and more information about the participants was provided in some of the studies, such as the stage of MS (relapsing-remitting or progressive) or their level on the EDSS (e.g., 2.5–5.5 or 4.0–6.0). Most of the studies used some sort of intervention design (*n* = 16) (such as randomized controlled trials, clinical trials or pretest-posttest studies) or cross-sectional design (*n* = 13). In 15 of the studies, no information was provided about the study design.

Based on critical appraisal the methodological quality of studies was acceptable. Studies with randomized controlled trial designs fulfilled all five criteria in three studies and seven studies met four criteria. In non-randomized studies one study fulfilled all five criteria and seven studies four criteria. For quantitative descriptive studies four studies met all five criteria, sixteen studies met four and three studies met three criteria.

### The focus of lower extremity health research in patients with MS

The research on lower extremity health focused primarily on two main areas: gait and lower extremity muscle strength (Table 2). The topics within the area of gait included walking endurance, gait parameters, foot placement during walking, balance and falls. The research on lower extremity muscle strength concentrated on analysing the muscle performance and capacity of the lower extremities during exercise or resistance training. There were single studies focusing on foot sensation, [50] foot vibration perception, [50, 60] neuropathic foot pain, [60] foot deformities, [75] lower limb oedema [80] and foot sudomotor function [60].

**Table 2:** Summary of the studies (*n* = 42) included on the review in the alphabetical order

Author, year, country	Aim	Design, sample	Data collection method	Main results	Quality appraisal (number of fulfilled criteria)
Boes et al. 2018, USA [45]	To determine whether a powered ankle-foot orthosis (AFO) that provides dorsiflexor and plantar flexor assistance at the ankle can improve walking endurance of persons with multiple sclerosis	Short-term intervention; *n* = 16 PwMS, mean age 54.6	Walking test	Powered ankle-foot orthosis did not improve endurance walking performance.	4
Boudarham et al. 2016, Switzerland [46]	To assess coactivation of agonist and antagonist muscles at the knee and ankle joints during gait in patients with multiple sclerosis, and to evaluate the relationship between muscle coactivation and disability, gait performance, dynamic ankle strength measured during gait, and postural stability.	Design not reported; *n* = 14 PwMS, mean age 51	3D-gait analysis	Coactivation was increased in the knee muscles during single support (proximal strategy) and in the ankle muscles during double support (distal strategy). The magnitude of coactivation was highest in the patients with the slowest gait, the greatest motor impairment and the most instability.	3
Bowser et al. 2015, USA [47]	To compare sit-to-stand biomechanics among three groups: people with multiple sclerosis who exhibit leg weakness, people with multiple sclerosis who have comparable strength to controls, and healthy controls.	Cross-sectional design; *n* = 21 PwMS divided in two groups: Leg weakness group *n* = 10, mean age 49.2 Comparable strength group *n* = 11, mean age 39.8	Lower extremity muscle strength	Persons with multiple sclerosis exhibiting leg weakness displayed decreased leg strength, greater trunk flexion, faster trunk flexion velocity and decreased knee extensor power compared to the other two groups, and slower rise times compared to controls.	3
Brincks et al. 2017, Denmark [48]	To examine the associations between postural balance, assessed by force platform stabilometry, and complex walking performance and maximal walking speed in mildly disabled persons with MS and healthy matched controls.	Cross-sectional study design; *n* = 13 PwMS, mean age 42	Walking performance, postural balance	Significant correlations were observed between sway area and Timed Up & Go and fastest safe walking speed in persons with MS.	4
Broekmans et al. 2010, Belgium [49]	To investigate the acute effects of long-term wholebody vibration on leg muscle performance and functional capacity in persons with multiple sclerosis.	A randomized controlled trial; *n* = 25 PwMS, mean age 47.9	Muscle maximal isometric and dynamic strength, strength endurance and speed of movement, function.	Leg muscle performance and functional capacity were not altered following 10 or 20 weeks of whole-body vibration.	4
Citaker et al. 2011, Turkey [50]	To investigate the relationship between the foot sensations and standing balance in patients with Multiple Sclerosis (MS) and find out the sensation, which best predicts balance.	Design not reported; *n* = 27 PwMS, mean age 36.74	Sensation, vibration sensation, standing on one-leg	Light touch-pressure, vibration, two-point discrimination sensations of the foot sole, and duration of one-leg standing balance were decreased in patients with MS. Sensation of the foot sole was related with duration of one-leg standing balance in patients with MS.	4
Citaker et al. 2013, Turkey [51]	To investigate the relationship between the lower extremity isometric muscle strength and standing balance in patients with MS.	Design not reported; *n* = 47 PwMS, mean age 36.98	Neurological disability, muscle strength, static one-leg standing balance	Hip flexor-extensor-abductor-adductor, knee flexor-extensor, and ankle dorsal flexor isometric muscle strength, and duration of one-leg standing balance were decreased in patients with MS. All assessed lower extremity isometric muscle strength and EDSS level were related duration of one-leg standing balance in patients with MS. All assessed lower extremity isometric muscle strength (except ankle dorsal flexor) was related with EDSS.	4
DeBolt et al. 2004, USA [52]	To examine the effects of an 8-week home-based resistance exercise program on balance, power, and mobility in adults with multiple sclerosis.	Pretest–posttest experimental group design; *n* = 29 female PwMS, mean age 50.3 *n* = 8 male PwMS, mean age 51.1 divided into exercise group and control group	Balance, mobility, leg power	Leg extensor power improved significantly in the exercise group, although measures of balance and mobility did not change.	4
Dodd et al. 2011, Australia [53]	To determine the effectiveness of progressive resistance training (PRT) for people with MS, focusing on improving the gait deficits common in this population.	Single blind randomized controlled trial; experimental group *n* = 36 PwMS, mean age 47.7 control group *n* = 35 PwMS, mean age 50.4	Walking endurance, maximal walking speed, muscle strength, muscle endurance, self-reported fatigue, health-related quality of life, muscle stiffness and spasm	No differences were detected in walking performance. PRT demonstrated increased leg press strength, increased reverse leg press strength, and increased muscle endurance of the reverse leg press.	4
Fritz et al. 2015, USA [54]	To determine the longitudinal relationships among quantitative measures of gait and balance in individuals with MS.	Longitudinal cohort study; *n* = 57 PwMS, mean age 45.86	Balance, walking, muscle strength, vibration	Increases in static posturography and reductions in dynamic posturography are associated with a decline in walk velocity and Timed 25-Foot Walk performance over time.	4
Gutierrez et al. 2005, USA [55]	To evaluate the effects of an 8-week lower-body resistance-training program on walking mechanics in persons with multiple sclerosis (MS)	Repeated-measures design; *n* = 8 PwMS, mean age 46.0	Kinematic gait parameters, isometric strength,3-min stepping, fatigue, self-reported disability	Resistance training increased significantly percentage of stride time in the swing phase, step length, stride length, and foot angle; and significantly decreased percentage of stride time in the stance and double-support phases, duration of the double-support phase, and toe clearance. Isometric leg strength improved in 2 of the 4muscle groups tested. Fatigue indices decreased, whereas self-reported disability tended to decrease following the training program.	4
Hayes et al. 2011, USA [18]	To assess the effects of a program of high-intensity RENEW exercise combined with standard exercises on lower extremity strength, mobility, balance, and fatigue in individuals with MS compared to a standard exercise program over 12 weeks.	A prospective, longitudinal, randomized intervention trial; *n* = 19 PwMS, mean age 49	Lower extremity strength, walking, balance, fatigue	No significant time effects or interactions were observed for strength, walking or balance.	4
Huisinga et al. 2013, USA [56]	To determine any differences in biomechanical gait parameters between patients with MS and healthy controls.	Quantitative evaluation; *n* = 31 PwMS, mean age 46.2	Joint torques and joint powers, walking	Reduced angular range, less joint torque, and reduced joint power were seen in patients with MS. Significant correlations between biomechanical gait parameters and EDSS score.	4
Jackson et al. 2008, USA [57]	To evaluate the acute effects of a brief exposure to WBV on quadriceps and hamstring muscle performance in persons with MS	Randomized, crossover study; *n* = 15 PwMS, mean age 54.6	Muscle torque	There were no significant differences in isometric torque production between the 2- and 26-Hz WBV conditions. There was also no significant difference between baseline torque values and those measured at one, 10, and 20 min after either vibration exposure.	5
Kalron 2017a, Israel [58]	To examine the relationship of obesity with walking and balance in people with multiple sclerosis.	A cross-sectional study; *N* = 436 PwMS divided into two groups: *n* = 258 normal weight, mean age 40.4 *n* = 178 obese, mean age 49.6	Spatiotemporal parameters of gait, postural control	Obese subjects walked significantly slower, with shorter step lengths and a wider step width. Thy walked a shorter distance on the 6-Minute Walk test and slower on the Timed 25-Foot Walk test.	5
Kalron 2017b, Israel [59]	To examine the relationship between variability of major spatio-temporal parameters of gait and falls, in PwMS with an expanded disability status scale score of 4.0 and 4.5	Cross-sectional study; *N* = 91 PwMS, mean age 48.0 divided into two groups: *n* = 50 fallers, mean age 48.8 *n* = 41 non-fallers mean age 46.7 years	Gait measures	The MS fallers presented a higher variability score in the step length and single support compared to participants in the non-fallers. Gait variability scores were significantly correlated with clinical walking tests.	5
Khan et al. 2018, Qatar [60]	To determine the prevalence and severity of neuropathic pain, sudomotor dysfunction and abnormal vibration perception in patients with MS	Design not reported; *n* = 73 PwMS, mean age 36.68	Disability, neuropathic pain, sudomotor function, vibration perception threshold	Patients with multiple sclerosis have evidence of sudomotor dysfunction and elevated vibration perception.	4
Kjolhede et al. 2015, Denmark [61]	To investigate the relationship between rate of force development (RFD) and maximal muscle strength of knee extensors and flexors and measures of functional capacity in PwMS	Clinical trial; *n* = 35 PwMS, mean age 43.3	Muscle strength, walking, stair climb, functional capacity	Rate of force development and maximal muscle strength correlated with functional capacity. Correlations were strongest for knee extensors and flexors of the weaker leg.	4
Larson et al. 2013, Greece [62]	To quantify bilateral differences in lower-limb performance and metabolism during exercise.	Design not reported: *n* = 8 PwMS, mean age 51.6	Muscle strength, walking	Individuals with MS had significant between-leg differences in leg strength, peak oxygen uptake, and peak workload.	4
McLoughlin et al. 2014, Australia [63]	To investigate the effect of walking-induced fatigue on lower limb strength and postural sway in people with moderately disabling MS.	Controlled study; *n* = 34 PwMS, mean age 49.1	Fatigue, postural sway, lower limb strength	Significant time by condition effects for all assessment measures indicated the six-minute walk induced fatigue with associated increases in postural sway and reductions in lower limb strength in people with MS.	4
Medina-Perez et al. 2016, Spain [64]	To examine the effects of 12 wk. of muscle power training on peak muscle power and maximal voluntary isometric contraction (MVIC) of knee extensors in patients with MS	Intervention study; *n* = 40 PwMS: divided into two groups *n* = 20 women with MS, mean age 42.8 *n* = 20 men with MS, mean age 44.0	Muscle strength	No significant changes in the control group from baseline to post-intervention evaluation. In contrast, the exercise group significantly increased MVIC and muscle power after the training.	5
Motl et al. 2012, USA [65]	To examine changes in walking function associated with combined exercise training consisting of aerobic, resistance, and balance activities in persons with MS who had recent onset of gait impairment	Design not reported; *n* = 13 PwMS, mean age 51.5	Walking, function	These results suggest that a moderately intense, comprehensive, combined exercise training program represents a rehabilitation strategy that is associated with improved walking mobility.	4
Neamtu et al. 2012, Romania [66]	To present morpho-functional limb aspects during gait at MS patients.	Design not reported; *n* = 13 PwMS, mean age 36	Biomechanical examination of the foot	Load and impulse had high values at MS patients; these patients displayed a significant right-left asymmetry during all the gait phases due to the lower propulsion force of the foot in stride.	3
Nogueira et al. 2013, Brazil [67]	To analyze the gait characteristics of MS patients in the absence of clinical disability	Case-control study; *n* = 12 PwMS, mean age 30.6	Disability, gait, perceived balance confidence, physical activity and fatigue	MS patients showed impairment of perceived fatigue, perceived of walking impact and perceived balance confidence, despite having no disability.	4
Pau et al. 2015, Italy [68]	To characterize the gait patterns of individuals with Multiple Sclerosis (MS) affected by spasticity using quantitative gait analysis.	Cross-sectional study; *n* = 38 PwMS, divided into two groups: *n* = 19 PwMS affected by lower limb spasticity, mean age 54.6 *n* = 19 PwMS not affected, mean age 47.1	Walking, range of motion, muscular activation	Spasticity originates a peculiar gait pattern characterized by reduced speed, cadence, stride length, swing phase and increased double support time, but they also reveal specific alterations in kinematics and muscular activation.	4
Pau et al. 2017, Italy [69]	To quantitatively assess the effect of 6 months of supervised adapted physical activity (APA i.e. physical activity designed for people with special needs) on spatio-temporal and kinematic parameters of gait in persons with Multiple Sclerosis (pwMS)	Randomized controlled trial; *n* = 22 PwMS, were randomly assigned: *n* = 11 intervention group, mean age 47.4 *n* = 11 control group, mean age 44.5	Gait analysis, range of motion	The training originated significant improvements in stride length, gait speed and cadence in the intervention group, while GPS and GVS scores remained practically unchanged. A trend of improvement was also observed as regard the dynamic ROM of hip, knee, and ankle joints.	4
Peruzzi et al. 2017, Italy [70]	To examine the effect of a virtual reality-based training on gait of people with multiple sclerosis.	Single blind randomized controlled trial *n* = 25 PwMS divided into two groups: *n* = 11 control group, mean age 42.0 *n* = 14 experimental group, mean age 43.6	Gait analysis, clinical motor tests, walking endurance and speed, mobility, balance, obstacle negotiation, disability	Subjects in both the groups significantly improved the walking endurance and speed, cadence and stride length, lower limb joint ranges of motion and powers, during single and dual task gait. Subjects in the experimental group also improved balance, as indicated by the results of the clinical motor tests.	5
Picelli et al. 2017, Italy [71]	To compare the clinical and ultrasonographic features of spastic equinus in patients with chronic stroke and multiple sclerosis	Observational study; *n* = 38 PwMS, mean age 53.2 *n* = 38 chronic stroke patients, mean age 61.4	Muscle spasticity	Affected calf muscles tone was significantly greater in patients with chronic stroke as well as spastic gastrocnemius muscle echo intensity. Affected ankle range of motion was significantly greater in patients with multiple sclerosis as well as spastic gastrocnemius muscle thickness.	4
Pike et al. 2012, France, Germany, Italy, Spain, the UK [44]	To evaluate the prevalence, severity and burden of walking and mobility problems (WMPs) in 5 European countries	Cross-sectional study, patient record-based study; *n* = 2171 PwMS, mean age 40.6	Mobility, walking	WMPs were regarded as the most bothersome symptom by almost half of patients. There was a clear, independent and strong directional relationship between severity of WMPs (subjective and objective) and healthcare resource utilisation. Patients with longer walking times (indicating greater walking impairment) were significantly more likely to require additional caregiver support, visit a variety of healthcare professionals including their primary care physicians and require more long-term non-disease modifying drug.	5
Ramdharry et al. 2006, UK [72]	To evaluate the effects of dynamic foot orthoses (DFO) on walking and balance performance in people with multiple sclerosis (MS).	Design not reported; *n* = 16 PwMS, age not reported	Walking speed, balance	Dynamic foot orthoses may increase sway and change centre of pressure position by altering foot alignment and/or plantar afferent stimulation.	5
Remelius et al. 2012, USA [73]	To investigate (1) whether previously observed changes in gait parameters in individuals with multiple sclerosis (MS) are the result of slower preferred walking speeds or reflect adaptations independent of gait speed; and (2) the changes in spatiotemporal features of the unstable swing phase of gait in people with MS.	Cross-sectional study; *n* = 19 PwMS, mean age 51.3	Gait analysis	Longer dual support time is part of a gait strategy in MS that is apparent even when controlling for the confounding effect of slower preferred speed.	4
Renfrew et al. 2019 UK [74]	To compare the clinical- and cost-effectiveness of ankle-foot orthoses (AFOs) and functional electrical stimulation (FES) over 12 months in people with Multiple Sclerosis with foot drop.	Multicentre, powered, non-blinded, randomized trial; *n* = 85 PwMS divided into *n* = 43 AFO group, *n* = 42 FES device group	Walking speed	Both devices demonstrated improvements in walking speed at 12 months, although there were no significant differences in their effects.	4
Rivera-Domingues et al. 1978, USA [75]	To study the prevalence of the foot deformities pes cavus and claw toes found in spastic spinal cord injury and multiple sclerosis patients and to discuss the pathogenesis of these foot deformities with the help of electromyography.	Design not reported; *n* = 20 PwMS, mean age 38 *n* = 80 spinal cord injury patients, mean age 43	Spasticity, foot assessment	Pes cavus and claw toes were found in two of 20 multiple sclerosis patients. All patients were spastic and had equinus deformity.	4
Rodgers et al. 1999, USA [76]	To examine the influence of an aerobic exercise program on lower extremity kinematics and kinetics during gait in patients with MS who demonstrate a range of disability	Design not reported; *n* = 18 PwMS, mean age 43.2	Range of motion, gait analysis	Hip passive range of motion increased. Mean walking velocity, cadence, and posterior shear push-off force decreased. During walking, maximum ankle dorsiflexion decreased and ankle plantarflexion increased. Results suggest this 6-mo training program had minimal effect on gait abnormalities.	4
Romberg et al. 2004, Finland [77]	To improve walking and other aspects of physical function with a progressive 6-month exercise program in patients with multiple sclerosis (MS).	Randomized controlled two-center intervention study; *n* = 47 PwMS, exercise group, mean age 43.8 *n* = 48 PwMS, control group, mean age 43.9	Walking speed, lower extremity strength, upper extremity endurance and dexterity, peak oxygen uptake, static balance.	Change between groups was significant in the 7.62 m and 500 m walk tests. In the 7.62 m walk test, 22% of the exercising patients showed clinically meaningful improvements.	4
Rusu et al. 2014, Romania [78]	Focus on biomechanical foot analyses of MS patients.	Clinical research; *n* = 48 PwMS, mean age 46.04	Biomechanical foot assessment	An instability left to right to be more evident in the swing phase and it influences the under the foot impulse for the next step and postural control.	4
Sandroff et al. 2013, USA [79]	To examine the associations among aerobic capacity, balance, and lower-limb strength asymmetries, walking performance, and gait kinematics in 31 persons with MS and 31 matched controls.	Design not reported; *n* = 31 PwMs, mean age not reported (range 18–54 years)	Peak aerobic capacity, muscular strength, balance, walking performance	Aerobic capacity, balance, and knee-extensor asymmetry were associated with walking performance and gait in persons with MS. Aerobic capacity and lower-limb strength asymmetries, but not balance, explained significant variance in walking performance and gait kinematics in the MS sample.	4
Solaro et al. 2006, Italy [80]	To evaluate the frequency of oedema of the lower limbs in multiple sclerosis(MS) patients utilizing a multidisciplinary approach	Design not reported; *n* = 205 PwMs, mean age 50.53	Assessment for presence of oedema or cutaneous complications.	Ninety-three patients (45%) showed oedema at the examination. EDSS, disease duration and disease course, but not gender, were statistically different between oedema and non-oedema patients	5
Sosnoff et al. 2011, USA [81]	To examine the hypothesis that persons with MS who had spasticity of the lower limbs would have more impairment of mobility and balance performance than persons with MS who did not have spasticity	Design not reported; *n* = 34 PwMS, mean age 57.5	Spasticity in muscles, walking speed, mobility, walking endurance, self-reported impact of MS on walking ability, balance	Fifteen participants had spasticity of the gastroc-soleus muscles based on modified Ashworth scale scores. The spasticity group had lower median EDSS scores indicating greater disability (*P* = 0.03). Mobility and balance were significantly more impaired in the group with spasticity compared to the group without spasticity.	4
Thoumie et al. 2002, France [82]	To evaluate the correlation between gait speed and strength in multiple sclerosis (MS) with particular regard to patients presenting with proprioceptive loss	Design not reported; *n* = 20 PwMS, mean age 42 years	Gait, muscle strength	Gait speed was reduced and strongly related to hamstring peak torque but not with quadriceps peak torque. In the patients with proprioceptive loss there was both a strong correlation between gait speed and hamstring torque and a significant correlation with quadriceps torque.	4
White et al. 2004, USA [83]	To evaluate the effect of an eight-week progressive resistance training programme on lower extremity strength, ambulatory function, fatigue and self-reported disability in multiple sclerosis (MS) patient	Experimental study; *n* = 8 PwMS, mean age 46	Isometric strength, walking, fatigue, disability	Knee extension, plantarflexion and stepping performance increased significantly. Self-reported fatigue decreased and disability tended to decrease following the training programme.	4
Yildiz et al. 2012, Switzerland [84]	To identify the relevance and impact of walking speed (WS) over a short distance on activities of daily living (ADLs) in patients with multiple sclerosis (MS).	Survey study; *n* = 112 PwMS, mean age not reported	Impact of MS on walking	Half of participants reported a high impact of MS on their general walking ability and their ability to increase WS over a short distance. Up to 53% of participants reported avoiding ADLs because of concerns about WS.	5

### Methods used to study lower extremity health in patients with MS

Lower extremity health was assessed using several different methods, most of which consisted of objective physical tests and gait analysis (Table 3). Mobility was assessed by conducting validated performance tests, such as a timed up-and-go test (*n* = 9), a timed 25-ft walk (*n* = 14) or a 500-m walk (*n* = 1). Aerobic capacity and endurance were measured with a 6-min walk test (*n* = 8) or a one-legged cycling test (*n* = 1). Gait was assessed from the perspective of gait biomechanics conducted using 3D or visual gait analysis methods. The gait analysis focused on walking speed (*n* = 15) including pelvic and hip kinematics, step width and walking velocity. Gait ability was assessed using a 2-min walk test (*n* = 5), a 10-m walk (*n* = 2), a stair climb test (*n* = 2) and Bessou’s locometer (*n* = 1), or by using the patient-reported Multiple Sclerosis Walking Scale (*n* = 6).

**Table 3:** Methods of assessing lower extremity health in patients with MS

Measurement focus	Data collection method	References
Gait (biomechanics)	3D or visual gait analysis of gait parameters (e.g. step width, swing, stride length)	[46, 54–56, 59, 67–70, 73, 76, 77, 79, 84]
	Fast walking speed	[48, 53]
Gait (ability)	2-min walk test	[49, 53, 58, 59, 61]
	10-m walk test	[18, 70]
	5-min walk test	[74]
	Stair climb test	[18, 61]
	Bessou’s locometer	[81]
	Multiple Sclerosis Walking Scale (self-reported)	[58, 65, 67, 72, 74, 81]
Mobility	Timed up-and-go test	[18, 48, 49, 52, 58, 59, 70, 81]
	Timed 25-ft walk	[44, 48, 49, 54, 58–61, 65, 74, 77, 79, 81, 83]
	500-m walk	[77]
Aerobic capacity and endurance	6-min walk test	[18, 45, 58, 62, 63, 70, 79, 81]
	One-legged cycling test	[62]
Physical activity	International Physical Activity Questionnaire	[67]
Muscle strength	Isokinetic dynamometry	[49, 55, 61]
	Isometric dynamometry	[18, 51, 57, 61–64, 77, 79]
	Dynamometer	[54, 82, 83]
	Sit to stand (5 times)	[61]
Muscle endurance	Number of repetitions on seated leg press	[53]
	3-min stepping test	[55]
Muscle stiffness and spasticity	Multiple Sclerosis Spasticity Scale	[53]
	Ashworth Scale	[50, 71, 81]
	Tardieu Scale	[71]
	Functional ambulatory score	[65]
	Resistance to passive range of motion	[75]
	Tendon reflexes	[75]
	Dynamic or passive range of motion	[68, 69, 76]
Balance	Stabilometry or posturography	[48, 54, 63, 72]
	Obstacle negotiation	[70]
	Timed standing on one leg	[50, 51]
	Static postural control	[58]
	Centre of pressure	[52, 79]
	Activities-specific Balance Confidence Scale	[67, 74, 81]
	Berg Balance Scale	[18, 49, 80, 81]
Specific foot assessments	Vibration perception	[50, 54, 60]
	Force distribution	[66, 78]
	Plantar pressure	[66, 78]
	Neuropathic pain	[60]
	Sudomotor function	[60]
	Oedema: Fovea’s sign, Stemmer’s sign	[80]
	Sensation: Semmes-Weinstein monofilament	[50]
	Foot deformities	[75]

Lower extremity muscle strength was assessed using clinical strength tests (Table 3). These included isokinetic (*n* = 3) or isometric (*n* = 9) dynamometry measures and the sit-to-stand test (*n* = 1). Three studies used a dynamometer to assess muscle strength without specifying the measurement focus. Muscle endurance was assessed by counting the repetitions conducted during a 3-min stepping test (*n* = 1) or on a seated leg-press machine. Muscle stiffness and spasticity were assessed using validated instruments, such as the Ashworth Scale (*n* = 3). Tests focusing on tendon reflexes, passive and dynamic range of motion in ankle and knee joints and resistance were also used to test muscle spasticity.

Balance was assessed using clinical tests and instruments (Table 3). Dynamic and static balance were measured with stabilometry and posturography (*n* = 4). Obstacle negotiation was used to measure balance in obstacle-crossing (*n* = 1). Postural control was measured by timing the duration of standing on one leg (*n* = 1). The centre of pressure in the sole of the foot was analysed in order to detect changes in plantar pressure (*n* = 2). The Activities-specific Balance Confidence Scale (*n* = 3) and the Berg Balance Scale (*n* = 4) were used as objective measures of balance problems in daily life.

Specific foot assessments (Table 3) focused on vibration perception in the sole of the foot (*n* = 3), foot biomechanics (*n* = 2), plantar pressure (*n* = 2), neuropathic pain (*n* = 1), sudomotor function (*n* = 1), oedema (*n* = 1), foot sensation (*n* = 1) and foot deformities (*n* = 1).

### Lower extremity problems in patients with MS

There were many lower extremity health problems in patients with MS (Table 4). Their lower extremities had undergone many biomechanical changes, which could be seen as a functional discrepancy of the load on the lower limbs and walking asymmetry [78].

**Table 4:** Lower extremity health problems in patients with MS

Lower extremity health problem	References
Decreased muscle strength in lower limbs	[46, 47, 51, 56, 62, 82]
Impaired balance	[48, 50, 51, 67]
Walking dysfunction	[44, 54, 59, 66, 67, 73, 78, 79, 82, 84]
Decreased pressure sensation	[50]
Decreased vibration sensation	[50, 60]
Sudomotor dysfunction	[60]
Oedema	[80]
Pes cavus	[75]
Claw toes	[75]
Spasticity	[68, 81]

Changes lower extremity biomechanics and muscle strength caused lower extremity problems which were affecting gait in many ways [48, 54, 56]. The lower extremity problems caused walking difficulties which were seen in the length of the step or the walking speed [44, 47, 48, 54, 73]. Walking and mobility problems and a limited range of motion in the lower limb joints were the most bothersome symptoms in patients with MS [44]. Perceived fatigue shortened the walking distance, [63, 67] and obesity was linked to a slower walking speed with shorter step lengths in patients with MS [58]. The altered lower extremity mechanics in these patients reflected a strength deficit compared with healthy controls during walking [56]. Patients with MS had increased coactivation in the knee and ankle muscles during the single or double support phases of the gait. This increased coactivation of muscles was associated with impaired postural stability and was a compensatory mechanism, where a patient with MS tried to walk as safely as possible [46]. Impaired postural balance resulted in decreased maximal walking performance and walking speed [48, 54].

Spasticity in the lower extremities was common (Table 4), [75] resulting in reduced balance [81] and creating an altered gait pattern characterised by reduced speed, rhythm, stride length and swing phase and increased double support time [68]. Muscle weaknesses in the lower limbs was prevalent in patients with MS and resulted in a slower sitting-to-standing time [47] and reduced balance [51]. Thus, reduced balance was related to slower walking velocity [54]. In the foot and ankle, pes cavus, claw toes, ankle equinus [75] and lower extremity oedema were prevalent [80]. Neuropathic pain and sudomotor dysfunction and elevated vibration perception of the feet were also common and associating with neurological disability caused by MS [60].

Some interventions aimed to support lower extremity health in patients with MS. Most of the intervention studies (*n* = 12) were focused on improving lower extremity muscle strength, [52, 53, 64, 83] walking, [53, 55, 65, 69, 70, 74, 76, 77, 83] balance, [51, 52] mobility [52] or kinematics in the lower extremities [76]. The interventions varied from various physical training methods to foot orthoses. Eight week progressive lower body resistance training significantly improved leg extensor power, [52] knee extension, plantarflexion and stepping performance, [83] and it also had positive effects on walking ability [55]. Supervised muscle power training for the knee extensors lasting 12 weeks improved peak muscle power and maximal voluntary isometric contraction of the knee extensors [64]. Physical activity program for 24 weeks consisting of aerobic and strength training resulted improvements is gait stride length, speed and cadence [65] and muscle endurance [69]. Aerobic training improved the passive range of movement in the hips, but it had only a minimal effect on gait abnormalities [76]. Virtual reality treadmill training for 6 weeks improved balance, walking endurance and gait kinematics [70]. Mixed evidence was found for the effects of foot orthoses [45, 72, 74] and whole-body vibration [49, 57] as interventions. Ankle-foot orthoses and functional electrical stimulation improved walking speed in patients with MS with foot drop [74]. Whereas dynamic foot orthoses increased body sway and changed the centre of pressure position [72]. Powered ankle-foot orthosis did not improve walking endurance of patients with MS [45].

## Discussion

Research on lower extremity health in patients with MS is an important and necessary aspect of rehabilitation research. This review highlights the importance of future research on the topic. On the basis of the results, it is evident that lower extremity research among patients with MS has focused strongly on gait and lower extremity muscle strength. Little emphasis has been placed on foot or ankle problems. It is understandable that the focus has been on lower extremity strength, as maintaining the ability to move is important for functional ability. However, sometimes relatively small changes in foot or ankle biomechanics and in foot health can affect walking ability; thus, more attention on this is needed in future.

The research methods applied in the studies were varied. Performance assessments were undertaken with validated and widely used methods such as timed 25-Foot Walk and 6-Minute Walk Test. The methods were strongly focusing on objective assessment of lower extremity health. Patients’ subjective perspective to lower extremity health was rarely under investigation. The use of qualitative methods such as interviews or written diaries could provide useful information about patients’ experiences of living with MS and lower extremity problems including how they manage in daily life in terms of walking, foot self-care and footwear which all are important while maintaining or promoting lower extremities. In addition, the use of information technology, such as active wristbands, could produce detailed follow-up information about level of physical activity and this information could be analysed against lower extremity health status.

The design of the research reviewed was traditional and in line with the research methods. Future studies could focus on more versatile research designs. Follow-up studies with many measurement points could provide evidence of how lower extremity health, specifically foot health, alters over time or in relation to MS disease activity. In addition, observational studies could provide important information how patients with MS care for their feet. With this kind of information, targeted interventions could be developed and tested in order to support patients with MS during the trajectory of the disease.

Many patients with MS have problems with the lower extremities of which the most widespread problems relate to reduced lower limb muscle strength, impaired balance and walking dysfunction. Moreover, pes cavus, claw toes, oedema and altered foot sensation were common. All these problems have a direct effect on quality of life [85]; thus, it is important to study them. In future, the use of wearable technology [86] or other new technologies could provide a new way to assess walking ability, balance and muscle strength in patients’ actual everyday circumstances. This kind of information could be used as the basis for future interventions and technological innovations.

One important aspect of lower extremity health is foot self-care, but there is limited evidence of how patients with MS manage this. Therefore, it is important to identify potential gaps in foot care knowledge and habits among patients with MS, which could be done by using, for example, information technology for communication. Moreover, the hindering and promoting factors for lower extremity health, including foot health, should be investigated in order to help develop interventions that support foot self-care.

Future research needs to investigate the foot problems experienced by patients with MS. There is limited evidence of foot health in patients with MS as only one study identified in this review focused directly on foot health revealing pes cavus and claw toes common in patients with MS [75]. The particular study focused only foot structural deformities omitting for example skin and nail problems and foot pain. Therefore in order to gain a full picture of foot health status in patients with MS a large scale foot health assessment studies are needed. Changes in foot and ankle biomechanics or muscle strength in the foot can alter gait [66] and decrease functional ability. More specifically, changes in foot biomechanics can cause changes in the skin, such as corns and calluses, which, because they may be painful, can lead to altered foot biomechanics increasing tissue stress and thus impacting on gait [87]. Therefore, a systematic and thorough foot and lower extremity assessment is needed to conduct with methods combining the assessment of mechanically-based pathologies in the skin, tissue stress and foot, ankle and lower extremity biomechanics.

Assessment of foot problems and timely provided care is important in patients with MS. If foot problems are left uncared they can have long-term consequences on a person’s overall health. To support identification of foot problems, there is a need to develop a systematic assessment framework for evaluating the impact of MS on the lower limbs and feet. This information could then be used in both clinical and research contexts. The impact of assessing foot problems, and the resulting foot-health interventions, needs to be evaluated in the clinical and research contexts. Patients with MS could benefit from an intervention study where the effectiveness of professional foot care and individual foot self-care outcomes are assessed in order to improve foot health.

### Strengths and limitations

This review has some strengths and limitations that must be considered when interpreting the results. The literature search was conducted in the Medline (PubMed), CINAHL and Cochrane Library databases. Both of these databases are scientific, international and widely used, and their coverage of research in the field of health sciences is comprehensive [88]. The literature search was conducted using a combination of MeSH terms (in Medline) and Major Headings (in CINAHL) related to MS and keywords related to lower extremity health. The use of MeSH or Major Headings was considered appropriate because MS is a universally agreed term and there is no synonym for the disease. On the other hand, several synonyms were used for lower extremity health in order to ensure a comprehensive approach to the topic. Despite the careful search term planning, some terms might have been missed; thus, the coverage of the review could have been limited. However, the search produced more than 400 hits, the vast majority of which focused on MS.

The major limitation of this review relates to the analysis of the studies that were included. The studies were heterogeneous and were conducted using different study designs. The variation in the research methods and the results posed challenges for the analysis. Instead of searching for in-depth information about lower extremity health in patients with MS, a decision to remain at the general descriptive level was made within the research team. This decision was in line with the general definition of systematic scoping reviews, which usually focus on identifying the potential size and scope of the available research literature [89]. Moreover, the heterogeneous nature and methodological diversity of the studies restricted the possibility to conduct meta-analysis. In future, a review focusing on studies with similar designs could be conducted in order to advance in-depth knowledge of lower extremity health in patients with MS. Despite these limitations, this review produced a summary and evidence of current research and demonstrated the need for further research.

## Conclusions

This systematic scoping review revealed that MS affects lower limb and foot health in ways that have the potential to affect patients’ daily life. However, the extent of these problems is unclear, and this is in due to a dearth of research that focuses on lower limb and foot health in this patient group. It is important to identify foot problems at an early stage to facilitate the provision of appropriate interventions in a timely manner. It is evident that there needs to be more focus on foot problems, both in the clinical environment and in the context of research.

## Data Availability

The datasets used and/or analysed during the current study are available from the corresponding author on reasonable request.

## Abbreviations

CINAHL: The Cumulative Index of Nursing and Allied Health Literature
EDSS: Expanded Disability Status Scale
MeSH: Medical Subject Headings
MS: Multiple sclerosis
PRISMA: The Preferred Reporting Items for Systematic Reviews and Meta-Analyses
